# On component-wise dissimilarity measures and metric properties in pattern recognition

**DOI:** 10.7717/peerj-cs.1106

**Published:** 2022-10-10

**Authors:** Enrico De Santis, Alessio Martino, Antonello Rizzi

**Affiliations:** 1 Department of Information Engineering, Electronics and Telecommunications, University of Roma “La Sapienza”, Rome, Italy; 2 Department of Business and Management, LUISS University, Rome, Italy

**Keywords:** Metric learning, Pattern recognition, Dissimilarity space, Euclidean embedding, Kernel methods, Pseudo-Euclidean embedding

## Abstract

In many real-world applications concerning pattern recognition techniques, it is of utmost importance the automatic learning of the most appropriate dissimilarity measure to be used in object comparison. Real-world objects are often complex entities and need a specific representation grounded on a composition of different heterogeneous features, leading to a non-metric starting space where Machine Learning algorithms operate. However, in the so-called unconventional spaces a family of dissimilarity measures can be still exploited, that is, the set of component-wise dissimilarity measures, in which each component is treated with a specific sub-dissimilarity that depends on the nature of the data at hand. These dissimilarities are likely to be non-Euclidean, hence the underlying dissimilarity matrix is not isometrically embeddable in a standard Euclidean space because it may not be structurally rich enough. On the other hand, in many metric learning problems, a component-wise dissimilarity measure can be defined as a weighted linear convex combination and weights can be suitably learned. This article, after introducing some hints on the relation between distances and the metric learning paradigm, provides a discussion along with some experiments on how weights, intended as mathematical operators, interact with the Euclidean behavior of dissimilarity matrices.

## Introduction

In the past few decades, the discipline of pattern recognition (PR), aiming to automatically discover regularities in data, focused most efforts in frameworks conceived to learn from examples, thus from observations. These frameworks exploit several machine learning techniques grounding on the data-driven approach (Bishop, 2006). Therefore, in these specific cases, the goal of a PR system is to find regularities in data aiming to reach good *generalization* capabilities by building a model from known observations (Hart, Stork & Duda, 2000). Thereby, at the basis of an automated PR pipeline there are the observations, that can be any type of measurements on real-world objects. Observations can be collected by hand or automatically by sensors. Furthermore, observations can be labeled and labels allow to distinguish the class or the category in which the object falls (Martino, Giuliani & Rizzi, 2018). Moving away from the “philosophical” problem among the differences between objects that live in the real world—a discussion that deserves a systematic and really interesting discussion—it can be stated that it is really difficult to enumerate all differences between two real-world objects, at least, at raw (atomic) level. So what we can reveal are the differences between the (physical or virtual) properties of two objects and tell whether they can be considered different. This task takes part to the PR process and deserves a thorough discussion. In the PR jargon, the problem is known as finding a good *representation* of objects, *e.g*., if the weight is important as a property defining the objects, it should be taken into account, otherwise the system should not consider the “weight” feature. A really interesting theoretical treatment, within the context of cognitive science, on how *natural properties* arise from generic objects in building a suitable representation is provided by Peter Gärdenfors with the theory of conceptual spaces (Gärdenfors, 2004).

A *representation* can exist in several forms, such as numbers, strings, graphs, images, spectra, time series, densities and similarities (Duin & Pękalska, 2012). Robert P. W. Duin states that (Pękalska & Duin, 2012): (i) “every real-world difference between objects that may play a role in the *human judgment* of their similarity should make a difference in the representation” and ii) “the representation of a real world object, *i.e*., the mapping from the object to its representation, should be continuous”. Hence, these prescriptions indicate that the representation should consider real-world properties judged as important and, furthermore, two similar objects should be similar in their representations too. On the top of a good representation, it is possible to train a myriad of learning algorithms capable to generate a model from data objects and, finally, to generalize towards previously unseen data. In fully supervised learning, the generalization process (classification) needs labeled examples, while unlabeled examples are used in unsupervised learning schemes (Jain, Murty & Flynn, 1999; Jain, Duin & Mao, 2000). As we will see, alongside the classical learning algorithms adopted in machine learning, it can be useful to learn a dissimilarity function tailored to the data at hand. This particular task belongs to the metric learning (ML) paradigm, a florid research field in PR (Lu et al., 2018; Bengio, Courville & Vincent, 2013).

As anticipated, many real-world objects in PR cannot be simply described by a set of measurements collected in real-valued vectors. In other words, the representation of objects may not easily start from a vectorial space and in this case the dissimilarity measure cannot be simply defined as a plain Minkowski distance, for example. In this case, a data structure, known as *dissimilarity matrix*, becomes clearly important. Thereby, in many cases, the core of a PR system is a custom-based dissimilarity measure, that is a way to measure the dissimilarity between samples of a given complex process that are described by a set of measurements that can (even simultaneously) involve real numbers, integers, vectors, categorical variables, graphs, spectra, histograms, unevenly objects/events sequences, time series *etc.* This happens when real-world objects possess a complex description arising from different intrinsic characteristics, each one caught by a suitable data structure. Thence, the overall dissimilarity can be chosen within the family of Euclidean distances, or within the general class of Minkowski distances. However, the structure of the given distance needs to take into account the different data structures. In technical literature, distances involving complex and possibly heterogeneous data structures are known as *component-wise* or *element-wise* distances (Jimenez, Gonzalez & Gelbukh, 2016) where, for each component, it is used a specific difference operator for the data structure at hand and, once collected, all of them are synthesized in a “template” distance that may have the Euclidean or Minkowski general form. In other words, distances are a function of the additive combination of the contributions of their components (Beals, Krantz & Tversky, 1968). A further generalization can be derived from the weighted Euclidean distance (WED) where a weight is associated to each component. In ML tasks, these weights can be suitably learned automatically, usually through an optimization procedure.

The WED is widely applied in PR problems such as in bioinformatics and personalized medicine (Hu & Yan, 2007; Martino et al., 2020; Di Noia et al., 2020), speech synthesis (Lei, Ling & Dai, 2010) or in the industrial field (Rao, 2012). For example, WED is used in clustering application dealing with side information (Xing et al., 2002). In fact, if a clustering algorithm, such as *k*-means, initially fails to find a meaningful solution for the problem at hand from the user point of view, the user is forced to manually tweak the metric until sufficiently good clusters are found. In Schultz & Joachims (2003), the authors present a method for learning a distance metric starting from relative comparison such as “A is closer to B than A is to C”. A similar application can be found in (Kumar & Kummamuru, 2008), where a local metric is learned.

Moreover, in many real-world problems dealing with complex systems, the starting space is not a vectorial space, being also often non-metric (*e.g*., in life sciences (Münch et al., 2020; Martino, Giuliani & Rizzi, 2018), engineering applications (D’urso & Massari, 2019; De Santis, Arnò & Rizzi, 2022; Kim, Lee & Kim, 2018) or cybersecurity (Granato et al., 2020, 2022)). Consequently, only the dissimilarity representation is available through the dissimilarity matrix, as stated above. Hence, in such cases, the dissimilarity matrix is a primitive data structure compared to the data matrix. As we will see in the following, a dissimilarity matrix ${\rm {\mathcal {D}}}$ is said to be “Euclidean” if it is perfectly (isometrically) embeddable in an Euclidean vector space in which the distances calculated in the latter are identical to the ones belonging to the entries of ${\rm {\mathcal {D}}}$ (De Santis, Rizzi & Sadeghian, 2018). Several standard classifiers are designed to work effectively on Euclidean vector spaces. Operating with a non-Euclidean (or even non-metric) dissimilarity matrix may cause some problems. As an example, a non-Euclidean distance matrix leads to a non-positive definite kernel and the quadratic optimization procedure used to train a support vector machine (Vapnik, 1998; Schölkopf, Burges & Smola, 1999) may thereby fail, not being fulfilled the Mercer conditions (Mercer, 1909; Duin, Pękalska & Loog, 2013; Pękalska & Duin, 2005). However, in order to train standard classifiers on this kind of data, some solutions can be found. The two main solutions are based either on considering the dissimilarity matrix as the starting vector space endowed with the standard Euclidean distance (dissimilarity space representation) or by adopting a suitable transformation of the dissimilarity matrix, leading to the Pseudo-Euclidean (PE) space (Pękalska & Duin, 2005; De Santis et al., 2018; De Santis, Rizzi & Sadeghian, 2018). In the current study, we will consider the last case. For the first case, the interested reader can be referred to Pękalska & Duin (2005).

It is well known that from dissimilarity data collected in form of a dissimilarity matrix ${\rm {\mathcal {D}}}$ it can be “reconstructed” the starting Euclidean space where the original data points lie (De Santis, Rizzi & Sadeghian, 2018). The reconstruction process (known as *embedding*) tries to generate the original vector space such that the distances are preserved as well as possible. Classical multi-dimensional scaling is an example of such embedding procedure (Borg & Groenen, 2005). For an Euclidean space all the distances are preserved and thus an Euclidean distance matrix can be embedded isometrically in an Euclidean space. For non-Euclidean distance matrices the Euclidean space is not “large enough” to embed the dissimilarity data even if they can be still embedded in the so called PE space (Goldfarb, 1984). The embedding procedure involves the eigendecomposition of the kernel matrix ${\bf G} = {\bf X}{{\bf X}^T}$, where ${\bf X}$ is the configuration matrix with data points organized as rows, also known as the Gram matrix (Horn & Johnson, 2013). The latter is a similarity matrix, obtainable through a suitable linear transformation of the dissimilarity matrix ${\rm {\mathcal {D}}}$.

In this work, we consider a class of PR problems involving a dissimilarity matrix ${\rm {\mathcal {D}}}$ deriving from a custom-based component-wise dissimilarity measure $d(x,y;{\bf w}):{\rm {\mathcal {F}}} \times {\rm {\mathcal {F}}} \to {{\rm {\mathbb R}}^ + }$. The following study is based on the characterization of *d* as a composite dissimilarity matrix of the form: $\sqrt {\overline {\bf{d}} _c^T{{\bf{W}}^T}{\bf{W}}{{\overline {\bf{d}} }_c}} $ computed as the ${\ell}$_2_ norm of the vector ${{\bar {\bf d}}_c}$ that collects the component wise (sub)-dissimilarity measures, of which the functional form is related to the specific features (*i.e*., data structure) within a suitable structured non-metric feature space ${\rm {\mathcal {F}}}$.

Within this framework, in this article we provide two characterizations. The first one tries facing the claim according to which the behavior of a general dissimilarity measure depends on the behavior of the component-wise (sub)-dissimilarities. Specifically, *d* generates an Euclidean dissimilarity matrix if the (sub)-dissimilarities ${d_{{{\rm {\mathcal {F}}}_j}}}$ are Euclidean. Therefore, the features ${{\rm {\mathcal {F}}}_j}$ over which it is induced a particular dissimilarity measure, *i.e*., a structural dissimilarity in the sense of Duin & Pękalska (2010), can influence the nature of the mathematical space where the learning algorithm works.

As concerns the second characterization, it is really interesting to arrange a mathematical interpretation of the weights pertaining the custom-based dissimilarity matrix, in particular wondering what is the influence of a weighting matrix ${\bf W}$ on the eigenspectrum of the underlying Gram matrix ${{\bf G}_w}$, that is the Gram matrix obtained from the weighted version of the dissimilarity matrix ${\rm {\mathcal {D}}}$. Unfortunately the relationship between the eigenvalues of ${\bf G}$ and ${{\bf G}_w}$ in a general case is not straightforward, being an open problem of mathematics (Zhang & Zhang, 2006; Fulton, 2000). It is approachable in particular cases of commuting matrices (in the case of matrices sharing a complete set of eigenvectors, *i.e*., *normal* matrices) or when one of the two is a scalar matrix, *i.e*., a matrix of the form ${\bf W} = k{\bf I}$. We will trace some results in the latter case.

Although this article aims at addressing these characterizations *via* a theoretical and mathematical viewpoint, the interested reader can find practical applications in the following articles. In De Santis et al. (2015), De Santis, Rizzi & Sadeghian (2018) a One-Class classification approach is used in the field of predictive maintenance and in the real-time recognition of faults in a real-world power grid, by processing heterogeneous information coming from smart sensors related to the power grid equipment and to the surrounding environment. The system exploits a clustering-genetic algorithm (GA) (Goldberg, 1989) approach where the weights of a custom based Euclidean dissimilarity measure are learned solving a suitable optimization problem. In De Santis et al. (2018), we addressed the problem of finding suitable representative elements in the dissimilarity space^1^ 1See Pękalska, Duin & Paclík (2006) for a discussion on the subject matter. in order to classify protein contact networks according to their enzymatic properties and in De Santis et al. (2022), the dissimilarity space embedding has been used to recognize signals pertaining to malfunctioning states of pressurization systems for high-speed railway trains. Finally, in Martino et al. (2020) the same problem of classifying protein contact networks according to their enzymatic properties has been solved by an hybridization of dissimilarity spaces and multiple kernel learning.

The degree of “non-metricity” and even of non-Euclidean behavior can be measured suitably with specific indexes obtained from the PE embedding such as the *Eigen-Ratio*, the *Negative Eigen-Fraction* and the *Non-Metricity Fraction*, each of which measures the non-Euclidean behavior, *e.g*., of a given dissimilarity matrix (Pękalska et al., 2006). Therefore, while the second question concerns the relation between ${\bf G}$ and ${{\bf G}_w}$, in the first characterization we are wondering what is the influence of dissimilarity weights on the Negative Eigen-Fraction, hence we are questioning on how it is possible to tune the non-Euclidean behavior of a custom-based dissimilarity matrix.

The article is organized as follows. In “Metric Learning” it is provided a brief review of the various ML paradigms treated in the literature. “On Metric Spaces and Dissimilarity Matrices” is a concise description of metric spaces and related dissimilarity matrices that serves as background. “The Weighted Euclidean Distance” is a deepening of the Euclidean distance structure and its weighted component-wise counterpart. “Characterization of a Composite Component-wise Dissimilarity” and “On the Presence of Weights in a Component-wise Dissimilarity and the Eigenspectrum of the Gram Matrix” sketch an experimental evaluation of the proposed principal investigations and, finally, “Conclusion” concludes the article.

## Metric learning

**Figure 1:**
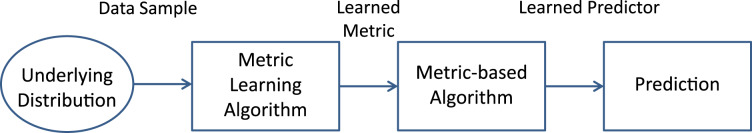
Scheme of the common process in Metric Learning. A metric is learned from data comingfrom a suitable distribution and plugged into a predictor (*e.g*., a classifier, a regressor, a recommendersystem, *etc*.). The predictor fed with the learned metric hopefully performs better than a predictorinduced by a standard, non-learned, metric (Bellet, Habrard & Sebban, 2013).

The ML problem is concerned with learning a distance function tuned to a particular task and has been shown to be useful when exploited in conjunction with techniques relying explicitly on distances or dissimilarities, such as clustering algorithms, nearest-neighbor classifiers, *etc.* For example, if the task is to asses the similarity (or dissimilarity) between two images with the aim of finding a match, *e.g*., in face recognition, we would discover a proper distance function that emphasizes appropriate features (hair color, ratios of distances between facial key-points, *etc.*). Although this task can be performed by hand, it is very useful to develop tools for learning automatically the subset of meaningful features for the problem at hand. In fact, as anticipated in “Introduction”, useful representations can be also learned. However, it is unquestionable that, at least on a theoretical level, representation learning must be taken separate from classification tasks as depicted in Fig. 1 and discussed in Bellet, Habrard & Sebban (2013).

The ML step can be conceived as a first step in the open-loop pipeline depicted in Fig. 1, to be performed before the model synthesis stage. Moreover, both tasks can be done together in the same system, representing an *advanced* closed-loop and automatic PR system. It is the case, for example, of feature selection and feature extraction techniques. The last procedures can be done manually, but if they are automated (*i.e*., optimized) they can be a building block of the classification system itself. There are many methodologies capable to learn a representation; some authors distinguish between neural learning, that is learning by means of deep learning techniques, and ML. Despite this distinction, in general, both approaches ground the learning procedure on optimization techniques. Neural learning is useful in finding a good feature space, while ML involves the learning of suitable manifold where data objects lie and where they can be well represented for solving the problem at hand.

**Figure 2:**
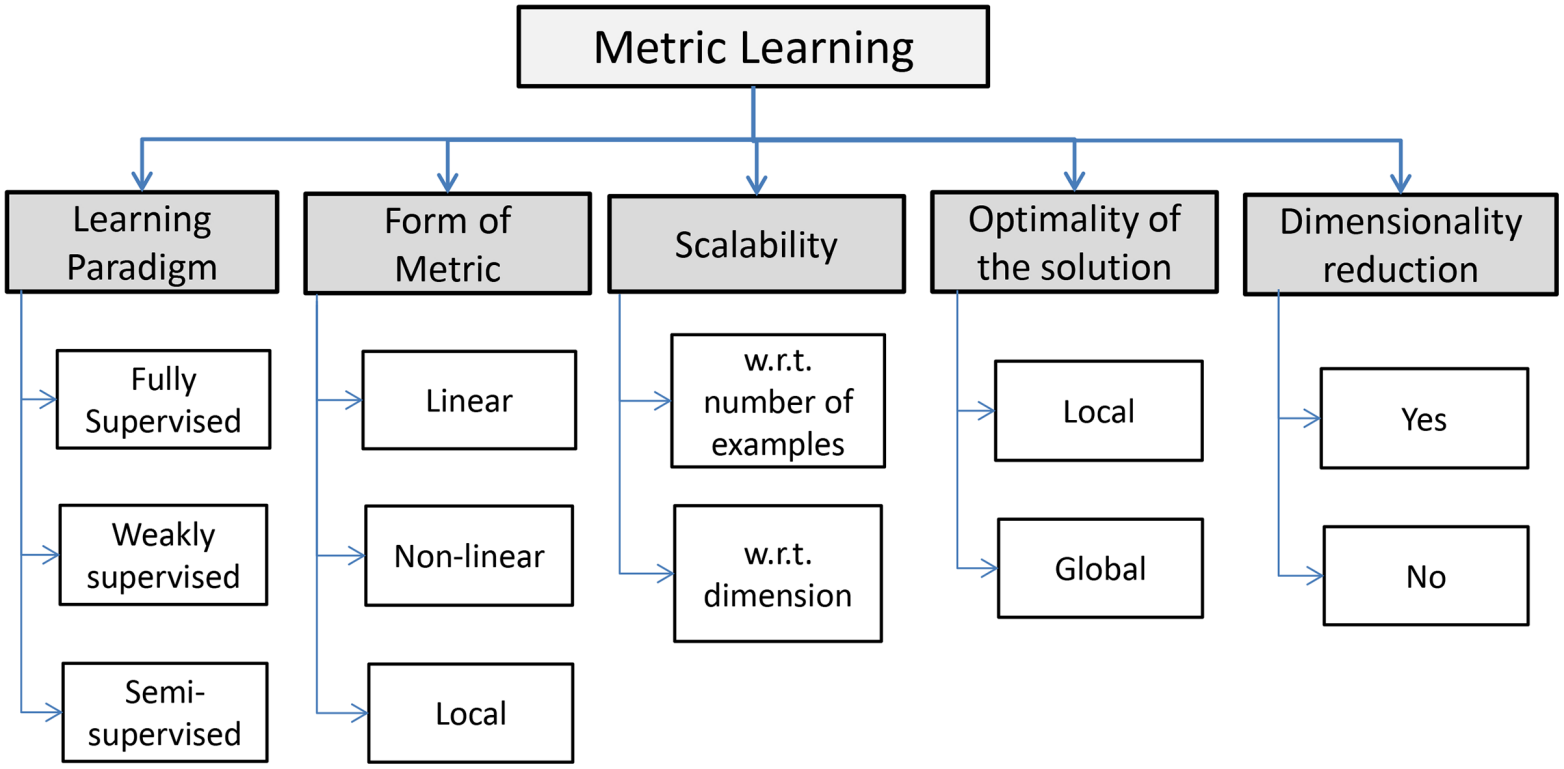
Five key properties of ML algorithms (Bellet, Habrard & Sebban, 2013).

Many declinations of ML are available and, according to Fig. 2, they can be resumed in three principal paradigms: *fully supervised*, *weakly supervised* and *semi supervised*. An informal formulation of the supervised ML task is as follows: given an input distance function $d({\bf x},{\bf y})$ between objects ${\bf x}$ and ${\bf y}$ (for example, the Euclidean distance), along with supervised information regarding an ideal distance, construct a new distance function ${\hat {d}}({\bf x},{\bf y})$ which is “better” than the original distance function (Kulis, 2012). Normally, fully supervised paradigms have access to a set of labeled training instances, whose labels are used to generate a set of constraints. In other words supervised ML is cast into pairwise constraints: the equivalence constraints where pairs of data points belong to the same classes, and inequivalence constraints where pairs of data points belong to different classes (Bar-Hillel et al., 2003; Xing et al., 2002). In weakly supervised learning algorithms we do not have to access to the label of individual training examples and learning constraints are given in a different form as side information, while semi-supervised paradigms do not use either labeled samples or side information. Some authors (*e.g*., Saul & Roweis (2003)) deal with unsupervised ML paradigms, sometimes called also manifold learning, referring to the idea of learning an underlying low-dimensional manifold^2^ 2A manifold is a topological space that resembles Euclidean space near each point. Hence a *n*-dimensional manifold has a neighborhood that is homeomorphic to the Euclidean space of dimension *n*. where geometric relationships (*e.g*., the distance) between most of the observed data are preserved. Often this paradigm coincides with the *dimensionality reduction* paradigm such as the well-known Principal Component Analysis (PCA) (Shlens, 2014; Giuliani, 2017) and the Classical Multi-Dimensional Scaling, based on linear relations. As concerns non-linear counterparts, it is worth taking note of embedding methods such as ISOMAP (Tenenbaum, De Silva & Langford, 2000), Locally Linear Embedding (Roweis & Saul, 2000) and Laplacian Eigenmap (Belkin & Niyogi, 2003). Other methods are based on information-theoretic relations such as the Mutual Information. Hence, the form or structure of the learned metric can be *linear*, *non-linear*, *local*. Linear ML paradigms are based on the learning of a metric in the form of a generalized Mahalanobis distance (Mahalanobis, 1936) between data objects, *i.e*., ${\rm {\mathcal {D}}}_{ij}^{\bf W} = \sqrt {{{\left( {{{\bf x}_i} - {{\bf x}_j}} \right)}^T}{{\bf W}^T}{\bf W}({{\bf x}_i} - {{\bf x}_j})} = \sqrt {{\rm \parallel }{\bf W}({{\bf x}_i} - {{\bf x}_j}){{\rm \parallel }_2}}$, where ${\bf M} = {{\bf W}^T}{\bf W}$ is a matrix with suitable properties that has to be learned. In other words, the learning algorithm learns a linear transformation ${\bf x} \to {\bf Wx}$ that better represents the similarity in the target domain. Sometimes, there are some non-linear structures in the available data that linear algorithms are unable to capture. This limitation leads to a non-linear ML paradigm, that can be based on the “kernelization” of linear methods or purely non-linear mapping methods. The last cases lead, for the Euclidean distance, to a kernelized version combining the learned transformation $\phi ({\bf x}):{{\rm {\mathbb R}}^m} \to {{\rm {\mathbb R}}^{\bar m}}$ with a Euclidean distance function with the capability to capture highly non-linear similarity relations, that is ${\rm {\mathcal {D}}}_{ij}^\phi = {\rm \parallel }(\phi ({{\bf x}_i}) - \phi ({{\bf x}_j})){{\rm \parallel }_2}$ (Kedem et al., 2012). Local metric refers to a problem where multiple local metrics are learned and often relies on heterogeneous data objects. In the last setting, algorithms learn using only local pairwise constraints. According to the scheme depicted in Fig. 2 the *scalability* of the solution is a challenging task, especially if we consider the growing of the availability of data in the Big Data era. The scalability could be important under the dataset dimension *n* and/or the dimensionality of data *m*. Finally, the intrinsic optimization task underlying the ML paradigm makes the optimality of the solution another important aspect. The latter, depends on the structure of the optimization scheme, that is, if the problem is convex or not (Boyd & Vandenberghe, 2004). In fact, for convex formulations it is guaranteed to reach a global maximum. On the contrary, for non-convex formulations, the solution may only be a local optimum.

## On metric spaces and dissimilarity matrices

### Definitions

The standard Euclidean space, as vector space, is highly structured from the algebraic viewpoint. Moreover, the Euclidean distance is experienced daily by human beings. PR problems do not involve necessary spaces with such an high level structure. Basically, from the PR point of view, a finite number of objects have to possess such properties that guarantee generalization, hence learning. The principal property is the “*closeness*” that relies on the notion of *neighborhood*, that is a primitive property applicable to general topological spaces (Deza & Deza, 2009). Furthermore, the metric properties that enrich the structure of primitive mathematical objects can be induced not only for a space but also for a set (*e.g*., the set of binary strings).

**Definition 1 **(Metric Space). Given a set *X*, a metric space is a pair (*X*, *d*), where *d* is a distance function $d:X \times X \to {\rm {\mathbb R}}_ + ^0$^3^ 3
${\rm {\mathbb R}}_ + ^0\equiv {\rm {\mathbb R}}_+\cup \{0\}$. such that the following conditions are fulfilled for ∀ *x*, *y*, *z* ∈ X^4^ 4Here it is not used the bold notation to indicate that *X* is a set of generic objects and not only a vector space. Hereinafter, the calligraphic notation (instead the bold one) will be used for the dissimilarity matrix ${{\rm {\mathcal {D}}}}$ and for the so-called centering matrix ${\rm {\mathcal {J}}}$.:

1. Reflexivity: *d* (*x, x*) = 0;

2. Symmetry: *d* (*x*, *y*) = *d* (*y, x*);

3. Definiteness: *d* (*x*, *y*) = 0 ⇒ *x* = *y*;

4. Triangular inequality: *d* (*x*, *z*) ≤ *d* (*x*, *y*) + *d* (*y*, *z*).

If all conditions are fulfilled *d* is properly said distance function. Conversely, if some conditions are weakened the space continues to have some structure and *d* is better known in PR as *dissimilarity*. For example, a space (*X*, *d*) that obeys only the reflexivity condition is known as *hollow space*; a hollow space^5^ 5The terminology is not unified, we refer to the one adopted in (Pękalska & Duin, 2005). that obeys the symmetry constraint is a *pre-metric space*; a pre-metric space obeying the definiteness is a *quasi-metric space*; a pre-metric space satisfying the triangle inequality is a *semi-metric space*.

**Definition 2 **(Metric for Dissimilarity Matrix ${\rm {\mathcal {D}}}$ (De Santis, Rizzi & Sadeghian, 2018)). Let ${\rm {\mathcal {D}}}$ be a symmetric dissimilarity matrix with positive off-diagonal elements *d_ij_* built on a set of n objects ${\rm {\mathcal {X}}} = \left\{ {{o_1},{o_2},...,{o_n}} \right\}$, where *d_ij_* = *f* (*o_i_*, *o_j_*) is a admissible measure of the dissimilarity between the objects *o_i_* and *o_j_*. ${\rm {\mathcal {D}}}$ is metric if the triangle inequality *d_ij_* + *d_jk_* ≥ *d_ik_* hold for all triplets $\left( {i,j,k} \right)$.

It is worth noting that if two objects are similar in a metric sense, every other object that has a relation with one will have a similar relation with the other. This property allows for one of the given objects being eligible for becoming a prototype in learning algorithms (Pękalska & Duin, 2005).

**Definition 3 **(Euclidean behavior (De Santis, Rizzi & Sadeghian, 2018)) A *n* × *n* dissimilarity (distance) matrix ${\rm {\mathcal {D}}}$ is Euclidean if it can be embedded in a Euclidean space $\left( {{{\rm {\mathbb R}}^m},{d_2}} \right)$, where *d*_2_ is the standard Euclidean distance, where *n* ≥ *m*. Hence, a configuration $\{ {{\bf x}_1},{{\bf x}_2},...,{{\bf x}_n}\}$ can be determined in ${{\rm {\mathbb R}}^m}$ such that ${d_2}\left( {{{\bf x}_i},{{\bf x}_j}} \right) = {\left\| {{{\bf x}_i} - {{\bf x}_j}} \right\|_2} = {d_{ij}}$ for all *i*, *j*.

A symmetric *n* × *n* matrix ${\rm {\mathcal {D}}}$ with zero diagonal is Euclidean *iff* ${\rm {\mathcal {D}}}_c^{*2} = {\rm {\mathcal {J}}}{{\rm {\mathcal {D}}}^{*2}}{\rm {\mathcal {J}}}$ is negative semi-definite. The quantity ${\rm {\mathcal {J}}} = {\bf I} - \displaystyle{1 \over n}{\bf 1}{{\bf 1}^T}$, where ${\bf I}$ is the identity matrix, denotes the centering matrix. If ${\rm {\mathcal {D}}}$ is Euclidean, it is also metric (Gower & Legendre, 1986).

Given a vector configuration $\{ {{\bf x}_1},{{\bf x}_2},...,{{\bf x}_n}\}$ in a Euclidean space $\left( {{{\rm {\mathbb R}}^m},{d_2}} \right)$ equipped with the standard inner product $\left\langle {{{\bf x}_i},{{\bf x}_j}} \right\rangle$ and organized in a *n* × *m* configuration matrix^6^ 6We are using the Machine Learning convention in which the *n* data vectors are organized as rows in the data matrix **X**, hence the Gram matrix is computed as **XX**^*T*^. In Linear Algebra, with data vectors organized as columns the Gram matrix is **X**^*T*^ **X** and **XX**^*T*^ is *n*-times the covariance matrix, if data vectors have zero mean.

${\bf X} = {\left[ {{\bf x}_1^T,{\bf x}_2^T,...,{\bf x}_n^T} \right]^T}$, the *n* × *n* Graminian (Gram) matrix ${\bf G}$, known in Machine Learning as *linear kernel* matrix, can be expressed by the inner product between all pairs of vectors ${{\bf x}_i},{{\bf x}_j}$ as ${\bf G} = {\bf X}{{\bf X}^T}$. Since the squared distance $d^{2}_{2}$ can be expressed in terms of inner product as $d_2^2\left( {{{\bf x}_i},{{\bf x}_j}} \right) = \left\langle {{{\bf x}_i} - {{\bf x}_j},{{\bf x}_i} - {{\bf x}_j}} \right\rangle = {\bf x}_i^T{{\bf x}_j}$, a linear relation between the Gram matrix ${\bf G}$ and the matrix of squared Euclidean distances ${{\rm {\mathcal {D}}}^{*2}}$ can be found. The relation between ${\bf G}$ and ${{\rm {\mathcal {D}}}^{*2}}$ is:

(1) $${\bf G} = - \displaystyle{1 \over 2}{\rm {\mathcal {J}}}{{\rm {\mathcal {D}}}^{*2}}{\rm {\mathcal {J}}}.$$

Conversely, the relation between ${{\rm {\mathcal {D}}}^{*2}}$ and ${\bf G}$ is:

(2) $${{\rm {\mathcal {D}}}^{*2}} = {\bf g}{{\bf 1}^T} + {\bf 1}{{\bf g}^T} - 2{\bf G},$$

where ${\bf g} = diag({\bf G})$.

Given a non-metric (pre-metric) or non-Euclidean symmetric dissimilarity matrix ${\rm {\mathcal {D}}}$, the eigendecomposition of the Gram matrix ${\bf G}$ by the factorization ${\bf G} = {\bf Q}\Lambda {{\bf Q}^T}$, where $\Lambda$ is a diagonal matrix of eigenvalues organized in descending order and ${\bf Q}$ is an orthogonal matrix of the correspondent eigenvectors, leads to the presence of negative eigenvalues and the indefiniteness of the corresponding Gram matrix ${\bf G}$. However an embedding is still possible by constructing a suitable space, *i.e*., the PE space, with a suitable inner product and norm^7^ 7A solution can be addressed by taking the absolute value of the negative eigenvalues, keeping in mind that the definition of the inner product generating the Gram matrix **G** changes consequently..

A generalization of the well-known Euclidean distance on a vector space ${\bf X} \subseteq {{\rm {\mathbb R}}^m}$ is the Minkowski distance.

**Definition 4 **(Minkowski distance). Given two vectors ${\bf x},{\bf y} \in {{\rm {\mathbb R}}^m}$ the Minkowski distance of order *p $\in$* (−∞, +∞) is defined as:

(3) $${d_p}({\bf x},{\bf y}) = {\left( {\sum\limits_{i = 1}^m {{\left| {{x_i} - {y_i}} \right|}^p}} \right)^{\displaystyle{1 \over p}}}.$$

Depending on the value of the *p* parameter this distance generalizes the Euclidean distance (*p* = 2) or the Manhattan distance (*p* = 1). Moreover, not for all values of *p* the distance is metric. For *p* = 2 it is trivially metric^8^ 8It is noted that the Minkowski distance can be induced by a norm only for *p* ≥ 1, i.e., the ${\ell}$*_p_* norm defined as: ||**x**||*_p_* = ${\left( {\sum\limits_{i = 1}^m {{\left| {{x_i}} \right|}^2}} \right)^{\displaystyle{1 \over p}}}$. being the standard Euclidean distance. For every value *p* ≥ 1 the Minkowski distance is metric, while there is a problem with the Triangular inequality for *p $\in$* (0,1). In fact, if we consider a dimension *m* = 2 and three points: $A = {\left[ {0,1} \right]^T},B = {\left[ {0,0} \right]^T},C = {\left[ {1,0} \right]^T}$ we have *d*_*p*_(*A*, *B*) = *d*_*p*_(*B*, *C*) = 1 and ${d_p}(C,A{) = 2^{\textstyle{1 \over p}}}$. Finally, ${d_p}(A,B) + {d_p}(B,C{) = 2 < 2^{\textstyle{1 \over p}}} = {d_p}(C,A)$, since *p* < 1. Hence the Triangular inequality is violated and *d*_*p*_ is quasi-metric (Pękalska & Duin, 2005).

### On embedding on a pseudo Euclidean space

As anticipated in De Santis, Rizzi & Sadeghian (2018), a PE space ${{\rm {\mathbb R}}^{(p,q)}}$, with signature $(p,q) \in {\rm {\mathbb N}}$, can be seen as the product of a real and imaginary valued Euclidean vector space ${{\rm {\mathbb R}}^p} \times i{{\rm {\mathbb R}}^q}$. In other words, a PE space is a direct product space ${{\rm {\mathbb R}}^p}\mathop \oplus \nolimits_\ {{\rm {\mathbb R}}^q}$ with an indefinite inner product that is positive in ${{\rm {\mathbb R}}^p}$ and negative in ${{\rm {\mathbb R}}^q}$. Hence, given two vectors ${\bf x},{\bf y}$ in this space the bilinear inner product can be defined as: ${\left\langle {{\bf x},{\bf y}} \right\rangle _{pe}}{\rm \buildrel\textstyle.\over= }  {{\bf x}^T}  {{\rm {\mathcal {J}}}_{pq}}{\bf y}$, where ${{\rm {\mathcal {J}}}_{pq}} = diag({{\bf 1}_p}, - {{\bf 1}_q})$. In the same way, the squared norm is defined as $\left\| {\bf x} \right\|_{pe}^2{\rm \buildrel\textstyle.\over= }{\left\langle {{\bf x},{\bf x}} \right\rangle _{pe}} = {{\bf x}^T}{{\rm {\mathcal {J}}}_{pq}}{\bf x}$, yielding the squared distance $\left\| {{\bf x} - {\bf y}} \right\|_{pe}^2{\rm \buildrel\textstyle.\over= }{\left\langle {{\bf x} - {\bf y},{\bf x} - {\bf y}} \right\rangle _{pe}} =  {\left( {{\bf x} - {\bf y}} \right)^T}{{\rm {\mathcal {J}}}_{pq}}\left( {{\bf x} - {\bf y}} \right)$, that can be also negative. The Gram matrix ${\bf G} = - \displaystyle{1 \over 2}{\rm {\mathcal {J}}}{{\rm {\mathcal {D}}}^{*2}}{\rm {\mathcal {J}}}$ is now expressed as:

(4) $${\bf G} = {\bf X}{{\rm {\mathcal {J}}}_{pq}}{{\bf X}^T},$$

where ${{\rm {\mathcal {J}}}_{pq}}$ is known as the *fundamental symmetry* in the PE space ${{\rm {\mathbb R}}^{(p,q)}}$. The isometric embedding can be found by a proper decomposition of **G** in a PE space:

(5) $${\bf G} = {\bf X}{{\rm {\mathcal {J}}}_{pq}}{{\bf X}^T} = {\bf Q}\Lambda {{\bf Q}^T} = {\bf Q}{\left| \Lambda \right|^{\displaystyle{1 \over 2}}}\left[ {\matrix{ {{{\rm {\mathcal {J}}}_{pq}}} &{} \cr {} &{\bf 0} \cr } } \right]{\left| \Lambda \right|^{\displaystyle{1 \over 2}}}{{\bf Q}^T},$$

where *p* + *q* = *k* and ${\left| \Lambda \right|^{\displaystyle{1 \over 2}}}$ is a diagonal matrix whose diagonal elements are the square root of the absolute value of the eigenvalues organized in descending order, first the positive ones and after the negative ones, followed by zeros. ${{\bf X}_k} = {{\bf Q}_k}\left| {{\Lambda _k}} \right|$ is the configuration of vectors in the PE space ${{\rm {\mathbb R}}^k} = {{\rm {\mathbb R}}^{(p,q)}}$ where *k* non-zero eigenvalues corresponding to *k* eigenvectors in ${\bf Q}$ are preserved.

Finally, the estimated PE covariance matrix ${\bf C}$ can be found as:

(6) $${\bf C} = \displaystyle{1 \over {n - 1}}{\bf X}{{\bf X}^T}{{\rm {\mathcal {J}}}_{pq}} = \displaystyle{1 \over {n - 1}}\left| {{\Lambda _k}} \right|{{\rm {\mathcal {J}}}_{pq}} = \displaystyle{1 \over {n - 1}}{\Lambda _k}{{\rm {\mathcal {J}}}_{pq}}.$$

Hence ${\bf X}$ is an uncorrelated representation and even if **C** is not positive definite in the Euclidean sense, it is positive definite in the PE sense and ${\bf X}$ can be interpreted in the general context of the indefinite kernel PCA approach.

## The weighted euclidean distance

Let be ${\bf X} \subseteq {{\rm {\mathbb R}}^{m \times n}}$ a *m* × *n* data matrix with *n* data objects, arranged as columns, ${{\bf x}_i} = {\left[ {{x_{1i}},...,{x_{mi}}} \right]^T} \in {{\rm {\mathbb R}}^m}$, where *m* is the dimension of the vectorial space where data points lie. The vector space is endowed with the standard scalar product ${{\bf x}_i} \cdot {{\bf x}_j} =  \left\langle {{{\bf x}_i},{{\bf x}_j}} \right\rangle =  {\bf x}_i^T{{\bf x}_j} = \sum\nolimits_{k = 1}^m {x_{ik}}{x_{jk}}{e_{ik}}{e_{jk}}$ while ${{\bf e}_i}$ is the *i*-th standard basis vector, *i.e*., a vector of all zeros except for the entry *k*, which has a 1. The Euclidean distance function^9^ 9The standard Euclidean distance is an instance of a more general family of distances parametrized by the exponent *p*, known as Minkowski distance family. See “Characterization of a Composite Component-wise Dissimilarity” for a short introduction. in such space equipped with the standard inner product $\left\langle { \cdot , \cdot } \right\rangle$ can be expressed as:

(7) $$\matrix{ {d({{\bf x}_i},{{\bf x}_j})} \hfill &{ = \sqrt {{{\left\| {{{\bf x}_i} - {{\bf x}_j}} \right\|}_2}} = \sqrt {\left\langle {{{\bf x}_i} - {{\bf x}_j},{{\bf x}_i} - {{\bf x}_j}} \right\rangle } = } \hfill \cr {} \hfill &{ = \sqrt {{{\left( {{{\bf x}_i} - {{\bf x}_j}} \right)}^T}\left( {{{\bf x}_i} - {{\bf x}_j}} \right)} = \sqrt {\sum\limits_{k = 1}^m {{\left( {{x_{ki}} - {x_{kj}}} \right)}^2}} ,} \hfill \cr }$$

where the elements ${{\rm {\mathcal {D}}}_{ij}} = d({{\bf x}_i},{{\bf x}_j}),i,j = 1,2,...,n$ form the entries of the *n* × *n* distance matrix ${\rm {\mathcal {D}}}$ between the objects ${{\bf x}_i}$ and ${{\bf x}_j}$ in ${\bf X}$.

Given a symmetric positive-definite matrix ${\bf M}$ with real-valued entries, *i.e*., ${\bf M} = {{\bf M}^T}$ and ${{\bf x}^T}{\bf Mx} \ge 0$, ${\bf x} \ne 0$, the entry ${\rm {\mathcal {D}}}_{ij}^{\bf M}$ of the WED matrix ${{\rm {\mathcal {D}}}^{\bf M}}$ can be expressed as:

(8) $$\matrix{ {{\rm {\mathcal {D}}}_{ij}^{\bf M} = {d_{\bf M}}({{\bf x}_i},{{\bf x}_j})} \hfill &{ = \sqrt {{{\left( {{{\bf x}_i} - {{\bf x}_j}} \right)}^T}{\bf M}({{\bf x}_i} - {{\bf x}_j})} = \sqrt {{{({{\bf x}_i} - {{\bf x}_j})}^T}{{\bf W}^T}{\bf W}({{\bf x}_i} - {{\bf x}_j})} = } \hfill \cr {} \hfill &{ = \sqrt {{{({\bf W}({{\bf x}_i} - {{\bf x}_j}))}^T}{\bf W}({{\bf x}_i} - {{\bf x}_j})} = } \hfill \cr {} \hfill &{ = \sqrt {\left\langle {{\bf W}({{\bf x}_i} - {{\bf x}_j}),{\bf W}({{\bf x}_i} - {{\bf x}_j})} \right\rangle } = \sqrt {{\rm \parallel }{\bf W}({{\bf x}_i} - {{\bf x}_j}){{\rm \parallel }_2}} ,} \hfill \cr {} \hfill &{} \hfill \cr }$$

where ${\bf M} = {{\bf W}^T}{\bf W}$ is the Cholesky decomposition (Strang, 1976) of matrix ${\bf M}$, that in the Hermitian general case, is found to be the decomposition of an Hermitian matrix in the product of a lower triangular matrix and its conjugate transpose.

**Figure 3:**
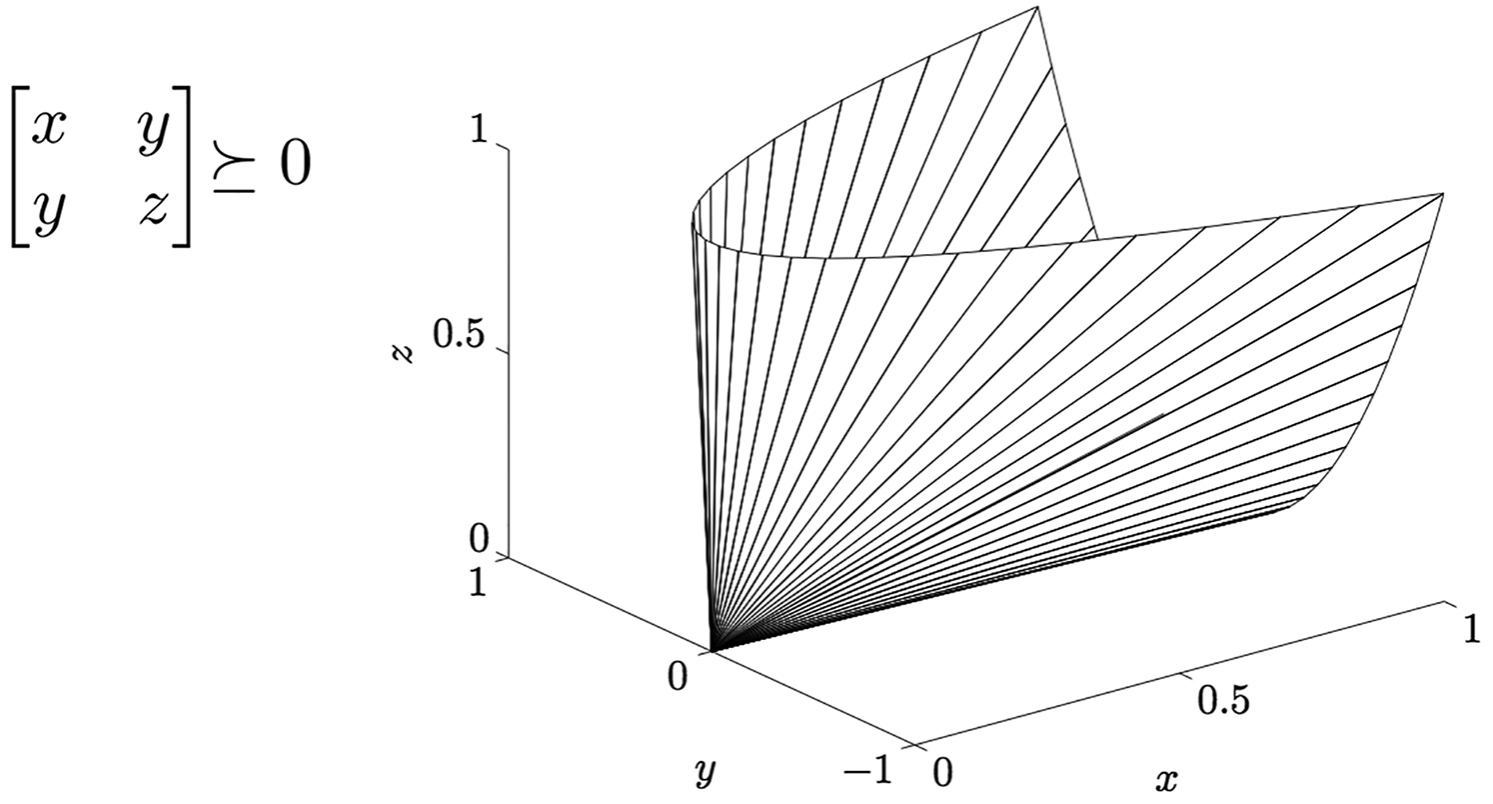
The cone S^m^_+_ of positive semi-definite 2 × 2 matrices.

In ML literature the distance in Eq. (8) is known as generalized Mahalanobis distance, a family of quadratic distances parametrized by a matrix ${\bf M} \in {\rm {\mathbb S}}_ + ^m$, where ${\rm {\mathbb S}}_ + ^m$ is the cone of symmetric positive semi-definite (PSD) *m* × *m* real-valued matrices–see Fig. 3. Note that ${\bf M} \in {\rm {\mathbb S}}_ + ^m$ ensures that the function ${d_{\bf M}}$ satisfies the properties of a pseudo-distance, *i.e*., $\forall {\bf x},{\bf y},{\bf z} \in {\bf X}$ holds:

1.
${d_{\bf M}}({\bf x},{\bf y}) \ge 0$ (non-negativity);
2.
${d_{\bf M}}({\bf x},{\bf x}) = 0$ (identity);
3.
${d_{\bf M}}({\bf x},{\bf y}) = {d_{\bf M}}({\bf y},{\bf x})$ (symmetry);
4.
${d_{\bf M}}({\bf x},{\bf z}) \le {d_{\bf M}}({\bf x},{\bf y}) + {d_{\bf M}}({\bf y},{\bf z})$ (triangular inequality).

The above properties hold trivially for a standard Euclidean space where ${\bf M} = {\bf I}$.

The matrix ${\bf W}$ can be seen as a linear operator that transforms the shape of the space where data points, *i.e*., data vectors, lie. Specifically ${\bf W}$ defines a suitable transformation (endomorphism) ${\rm {\mathcal {V}}} \to {\rm {\mathcal {V}}}$ of the (abstract) space ${\rm {\mathcal {V}}}$ spanned by rows vector of ${\bf X}$ in itself: given a vector ${\bf x}$ in the starting space *S*_1_, the matrix ${\bf W}$ maps this vector in a new vector ${{\bf x}_w} = {\bf Wx}$ that lies in the space *S*_2_, where *S*_1_ and *S*_2_ are isomorphic to ${\rm {\mathcal {V}}}$^10^ 10If **M** = **W**^*T*^**W** is strictly positive definite, **W** is a triangular matrix with no 0’s entry in the principal diagonal, hence it is invertible and we have **W**^−1^**x***_w_* = **x** and **W**^−1^**y**_*w*_ = **y**. Moreover, if an Hermitian matrix is positive semi-definite the Cholesky decomposition is still available, having the possibility of 0’s entries on the diagonal of **W**. Finally the Cholesky decomposition is unique when M is positive definite, while it is not true when it is positive semidefinite (Golub & Van Loan, 2012).. In the new transformed space the inner product becomes the standard inner product $\left\langle { \cdot , \cdot } \right\rangle$, *i.e*., $\left\langle {{{\bf x}_w},{{\bf y}_w}} \right\rangle = \left\langle {{\bf Wx},{\bf Wy}} \right\rangle = {\bf x}_w^T{{\bf y}_w} = ({\bf Wx}{)^T}({\bf Wy}) = {{\bf x}^T}{{\bf W}^T}{\bf Wx}$. The arrival space is endowed with a squared norm given by $\left\langle {{{\bf x}_w},{{\bf x}_w}} \right\rangle = \left\langle {{\bf Wx},{\bf Wx}} \right\rangle = \left\| {{\bf Mx}} \right\|_2^2$, being ${\bf M} = {{\bf W}^T}{\bf W}$^11^ 11We can assert that each space of vectors **x** comes with its dual-space of *linear functionals* **w**^*T*^. In the scalar product **w**^*T*^ x, **w**^*T*^ acts linearly upon vectors x and y, *i.e*., **w**^*T*^ (*λ***x**+ *µ***y**) = *λ***w**^*T*^ x+ *µ***w**^*T*^ y. At the same time **x** acts linearly upon **v**^*T*^and **w**^*T*^, *i.e*., (*λ***v***^T^* + *µ***w**^*T*^)**x** = *λ***v**^*T*^**x** + *µ***w**^*T*^**x**. So the linear functionals **w**^*T*^ form a vector space Dual or Conjugate to the space of vectors **x**. Each space is dual to the other, and they have the same finite dimension..

**Observation 1.** The weighted distance ${d_{\bf M}}({\bf x},{\bf y}) = d({\bf x},{\bf y},{\bf M})$ with ${\bf M} = {{\bf W}^T}{\bf W}$ equals ${d_{\bf I}}({{\bf x}_w},{{\bf y}_w}) = d({{\bf x}_w},{{\bf y}_w},{\bf I})$ where ${{\bf x}_w} = {\bf Wx}$ and ${{\bf y}_w} = {\bf Wy}$ and ${\bf I}$ is the identity matrix.

*Proof*. The proof follows by the same algebraic manipulation of Eq. (8).

Let ${\bf M} = {{\bf W}^T}{\bf W}$, ${{\bf x}_w} = {\bf Wx}$ and ${{\bf y}_w} = {\bf Wy}$. It holds that:

(9) $$\matrix{ {d\ \left( {{\bf x},{\bf y},{\bf M}} \right)} \hfill &{ = \sqrt {{{\left( {{\bf x} - {\bf y}} \right)}^T}{\bf M}\left( {{\bf x} - {\bf y}} \right)} = \sqrt {{{\left( {{\bf x} - {\bf y}} \right)}^T}{{\bf W}^T}{\bf W}\left( {{\bf x} - {\bf y}} \right)} = } \hfill \cr {} \hfill &{ = \sqrt {{{\left( {{\bf W}\left( {{\bf x} - {\bf y}} \right)} \right)}^T}{\bf W}\left( {{\bf x} - {\bf y}} \right)} = \sqrt {{{\left( {{\bf Wx} - {\bf Wy}} \right)}^T}\left( {{\bf Wx} - {\bf Wy}} \right)} = } \hfill \cr {} \hfill &{ = \sqrt {{{\left( {{{\bf x}_w} - {{\bf y}_w}} \right)}^T}\left( {{{\bf x}_w} - {{\bf y}_w}} \right)} = d({{\bf x}_w},{{\bf y}_w},{\bf I}).} \hfill \cr }$$

□

**Figure 4:**
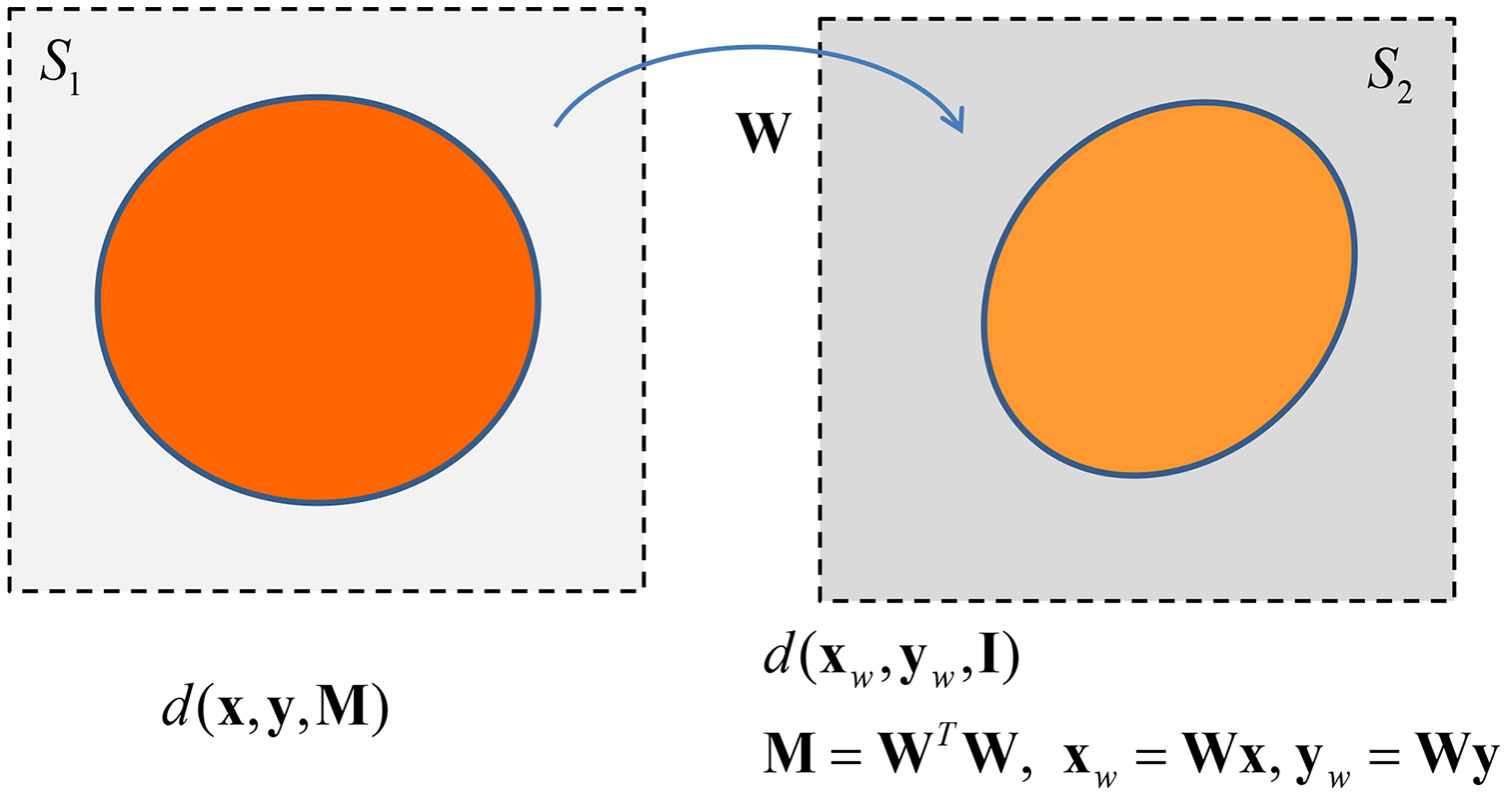
The transformation between Euclidean spaces by the linear operator W.

The matrix ${\bf W}$ is an instance of an operator that defines a rotation and a scaling of the objects upon it operates. ${\bf W}$ maps a circle in the unweighted Euclidean space in an ellipse in the weighted Euclidean space–see Fig. 4. Hence we can state the following theorem.

**Theorem 1.** Applying a transformation ${\bf W}$ to all point of a circle of radius *r* the resulting points form an ellipse whose center is the same as the circle and length of its axes equals *r* times twice the square root of eigenvalues of ${\bf M} = {{\bf W}^T}{\bf W}$.

*Proof*. An interesting demonstration can be found in Kurniawati, Jin & Shepherd (1998). □

The weight matrix ${\bf M}$ can be decomposed in its rotation and scaling components by means of the eigendecomposition operation. Specifically, by decomposing ${\bf M} = {\bf QD}{{\bf Q}^T}$ where ${\bf Q}$ is an orthogonal matrix with normalized column vectors, that is ${{\bf Q}^T}{\bf Q} = {\bf I}$ and ${{\bf Q}^T} = {{\bf Q}^{ - 1}}$, and ${\bf D}$ is a diagonal matrix^12^ 12The eigendecoposition results in a safe operation because **M** is a (square) real symmetric matrix, furthermore it can be demonstrated (spectral theorem (Strang, 1976)) that **M** is diagonalizable by the matrix of its eigenvectors, *i.e*., from the fundamental equation about the eigendecomposition: **MQ** = **QD**, by multiplying on the left both sides by **Q**^*T*^ we have **Q**^*T*^**MQ** = **D**. Finally the symmetry property leads to a set of real-valued eigenvalues and, being **M** a positive definite matrix, all the eigenvalues are positive.. ${\bf D}$ contains the eigenvalues λ_1_,λ_2_,…,λ_*m*_ (organized in decreasing order) that are the scaling factors, while ${\bf Q}$ is the rotation operator matrix that leaves unchanged the (squared) norm of vectors, that is $\left\| {{\bf Qx}} \right\|_2^2 =  \left\langle {{\bf Qx},{\bf Qx}} \right\rangle = {{\bf x}^T}{{\bf Q}^T}{\bf Qx} = {{\bf x}^T}{\bf x} = \left\| {\bf x} \right\|_2^2$ (Strang, 1976).

At this point it is possible to express the WED in terms of the above eigendecomposition:

(10) $$\matrix{ {d\left( {{\bf x},{\bf y},{\bf M}} \right)} \hfill &{ = \sqrt {{{\left( {{\bf x} - {\bf y}} \right)}^T}{\bf M}\left( {{\bf x} - {\bf y}} \right)} = \sqrt {{{\left( {{\bf x} - {\bf y}} \right)}^T}{{\bf W}^T}{\bf W}\left( {{\bf x} - {\bf y}} \right)} = } \hfill \cr {} \hfill &{ = \sqrt {{{\left( {{\bf x} - {\bf y}} \right)}^T}{\bf QD}{{\bf Q}^T}\left( {{\bf x} - {\bf y}} \right)} = \sqrt {{{\left( {{{\bf Q}^T}{\bf x} - {{\bf Q}^T}{\bf y}} \right)}^T}{\bf D}\left( {{{\bf Q}^T}{\bf x} - {{\bf Q}^T}{\bf y}} \right)} .} \hfill \cr }$$

From Eq. (10) it follows that $d\left( {{\bf x},{\bf y},{\bf M}} \right)$ can be expressed, through the eigendecomposition of the weighting matrix ${\bf M}$, with another weighted distance with weights given by the eigenvalues matrix ${\bf D}$. This new distance takes into account new vectors: ${{\bf Q}^T}{\bf x} = {\hat {\bf x}}$ and ${{\bf Q}^T}{\bf y} = {\hat {\bf y}}$ that are the rotated counterparts of original vectors ${\bf x}$ and ${\bf y}$. In other words, the two vectors ${\hat {\bf x}}$ and ${\hat {\bf y}}$ are the rotated, but not scaled, version of ${{\bf x}_w}$ and ${{\bf y}_w}$ that originate both in space *S*_2_. It can be demonstrated that the length of the axis of the ellipsoid in the direction of *i*-th eigenvalue *λ*_*i*_ is equal to: $\sqrt {d\left( {{\bf x},{\bf y},{\bf M}} \right)/{\lambda _i}}$.

**Figure 5:**
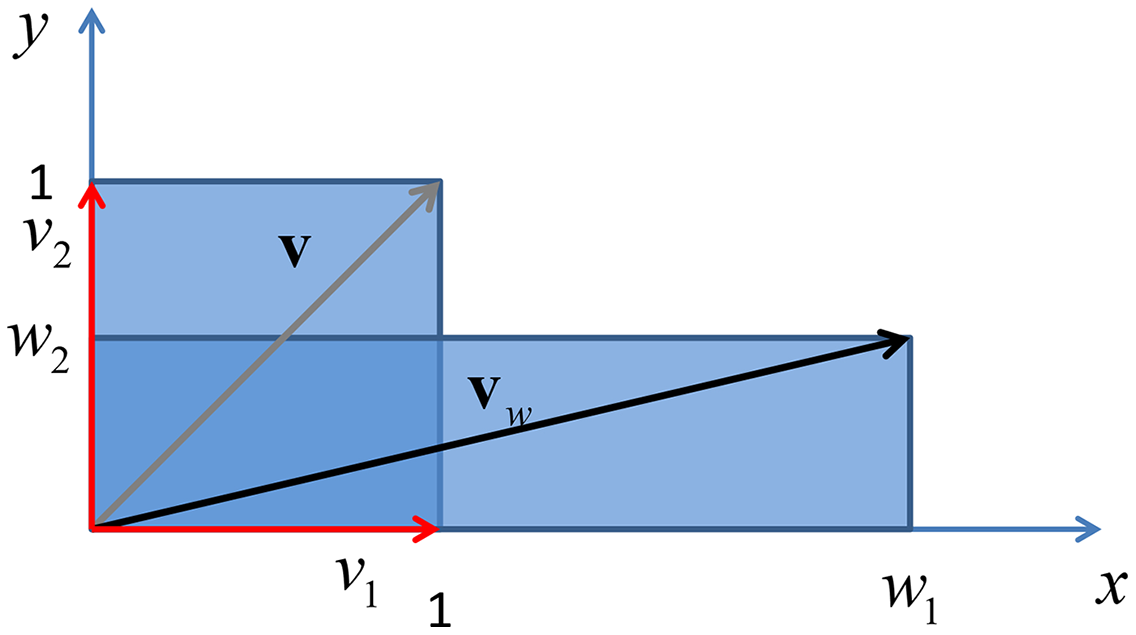
Vertical shrink and horizontal stretch of a unit square through a transformation induced by adiagonal matrix with real positive entries.

Finally if the weighting matrix ${\bf M}$ is a diagonal matrix, with real entries, the above eigendecomposition reduces to ${\bf M} = {\bf ED}{{\bf E}^T}$ where ${\bf E}$ is the eigenvector matrix ${\bf E} = \left[ {{{\bf e}_1},{{\bf e}_2},...,{{\bf e}_m}} \right]$, whose columns contains the standard basis in ${{\rm {\mathbb R}}^m}$ with the property ${\bf e}_i^T{{\bf e}_j} = {\delta _{ij}}$, where *δ*_*ij*_ is the Kronecker delta. In this case the matrix ${\bf E}$ represents the identity element of the rotation operator, leaving vectors in the original place, while they are scaled by a factors given by the entries of the diagonal of ${\bf M}$, being the eigenvalues of a diagonal matrix the diagonal entries of the same matrix. An example of this phenomenon is given in Fig. 5.

## Characterization of a composite component-wise dissimilarity

When the PR problem at hand deals with heterogeneous measures on objects and these measures are both structurally and semantically different (graphs, time series, images, real numbers, *etc.*), a composite dissimilarity measure can be useful, for example in clustering applications. The dissimilarity measure is a combination of (sub)-dissimilarities suitably defined depending on the nature of the data.

Before constructing a toy composite dissimilarity measure, it is worth to mention the following corollary valid when a dissimilarity measure is computed by combining the dissimilarities pertaining to all of the *m* attributes separately. In fact, given *m* features, a dissimilarity measures can be computed as: $d(x,y) = \sum\nolimits_{i = 1}^m f({x_i},{y_i})$, where *f* (*x*_*j*_, *x*_*j*_) = 0 and *f* (*x*_*j*_, *y*_*j*_) = *f* (*y*_*j*_, *x*_*j*_) ≥ 0 for all *j*. The corollary states:

**Corollary 1.** Let $x,y \in {{\rm {\mathbb R}}^m}$. Then $d(x,y) = \sum\nolimits_{i = 1}^m f({x_i},{y_i})$ is metric *iff* *f* is metric on ${\rm {\mathbb R}}$.

*Proof*. The proof can be done considering that *f* is non-negative, symmetric and it holds that *f* (*s*, *s*) = 0 for $s \in {\rm {\mathbb R}}$, then the first three axioms about metric spaces, *i.e*., reflexivity, symmetry and definiteness, are fulfilled. Furthermore, since *d* is metric *d*(*x*, *y*) + *d*(*y*, *z*) ≥ *d*(*x*, *z*) holds for all *x*, *y*, *z*. If we consider *x*_*j*_ = *c*_*x*_, *y*_*j*_ = *c*_*y*_, *z*_*j*_ = *z*_*x*_, for all *j* and some constants *c*_*x*_, *c*_*y*_, *c*_*j*_ the Triangle inequality for *d* reduces to *f*(*x*_*c*_, *y*_*c*_) + *f*(*y*_*c*_, *z*_*c*_) ≥ *f*(*x*_*c*_, *z*_*c*_). The ⇒ proof is trivial. □

Moreover, it can be demonstrated (Gower & Legendre, 1986) that, at least for the Euclidean case (*p* = 2 in the Minkowski distance definition), if $f:X \times Y \to {\rm {\mathbb R}}_ + ^0$ is a function, then $d(x,y) = \sum\nolimits_{i = 1}^m f({x_i},{y_i})$ is metric *iff* ${d^{'}}(x,y) = \sum\nolimits_{i = 1}^m {\left[ {f{{({x_i},{y_i})}^2}} \right]^{1/2}}$ is metric.

Now we show a demonstration of the following claim valid for a composite dissimilarity measure, making use of Def. 3 that characterizes the Euclidean behavior for dissimilarity matrices and Def. 2 for metric behavior.

**Claim 1.** Given two general objects $x,y \in {\rm {\mathcal {H}}}$, where ${\rm {\mathcal {H}}}$ is a generic feature space, and a component wise custom-based dissimilarity $d(x,y) = \sqrt {{{(x{\rm \ominus }y)}^T}(x{\rm \ominus }y)} =  \sqrt {\sum\nolimits_{i = 1}^m {{({x_i}{\rm \ominus }{y_i})}^2}}$, then if at least one component-wise dissimilarity is not Euclidean the dissimilarity matrix that arises from *d* applied on object within ${\rm {\mathcal {H}}}$, is not Euclidean.

As stated in “On Metric Spaces and Dissimilarity Matrices”, the expression “non-Euclidean” means that there is no set of vectors in a vector space of any dimensionality for which the Euclidean distances between the objects are identical to the given ones (Duin & Pękalska, 2010). We show now how Claim 1 can be demonstrated with a constructive example. Let ${\bf x} = ({x_1},{x_2},...{x_k},{x_{k + 1}},...{x_m})$ and ${\bf y} = ({y_1},{y_2},...{y_k},{y_{k + 1}},...{y_m})$ be two objects in a vectorial space ${{\rm {\mathcal {H}}}_v}$. We define a set of component-wise dissimilarities induced for the first *k* components such as $f_j^{cw}({x_j},{y_j}) = \left| {{x_j} - {y_j}} \right|,j = 1,2,...,k$ and a single component-wise dissimilarity induced for the remaining *m* − *k* components such as ${f^p}({x^{s = k + 1,...,m}},{y^{s = k + 1,...,m}}) = {\left( {\sum\nolimits_{s = k + 1}^m {{\left| {{x_s} - {y_s}} \right|}^p}} \right)^{\textstyle{1 \over p}}}$. In other words we divide the starting space ${{\rm {\mathcal {H}}}_v}$ as the Cartesian product (Strang, 1976) between two sub-spaces, the space ${{\rm {\mathcal {H}}}_{cw}}$ generated from the first *k* components, in which the component-wise dissimilarities are computed as *f*^*cw*^ and ${{\rm {\mathcal {H}}}_p}$ where the dissimilarity is computed as the Minkowski distance *f*^*p*^ applied to the last *m* − *k* components. Finally, the overall dissimilarity between two objects, say ${\bf x},{\bf y}$ is induced by the *ℓ*_2_ norm in the following way:

(11) $$\hat d({\bf x},{\bf y}) = \sqrt {{{\left( {{f^{cw}} \oplus {f^p}} \right)}^T}\left( {{f^{cw}} \oplus {f^p}} \right)} ,$$

where (*f*^*cw*^ ⊕ *f*^*p*^) is the vector of dimension *k* + *l* constructed by the concatenation of the two (sub)-dissimilarities *f*^*cw*^_*j*_, *f*^*p*^, *j* = 1,2,…,*k*.

To evaluate the validity of the Claim 1 the dissimilarity in Eq. (11) is computed on a sample drawn from a multi-variate Gaussian distribution with dimension *m* parameterized as: *m* = *k* + *l*, where *k* is maintained fixed without loss of generality, and *l* is varied. It is noted that the *p* parameter controls the nature of the Minkowski distance, making the (sub)-dissimilarity *f*^*p*^ metric or not metric (and even non-Euclidean^13^ 13The Euclidean property defined in Def. 3 is more restrictive than the metric property. Thereby, there are spaces that are metric but non-Euclidean. The opposite does not hold.) depending on the value of *p* as demonstrated above, such that for *p* ≥ 1 it is metric.

**Figure 6:**
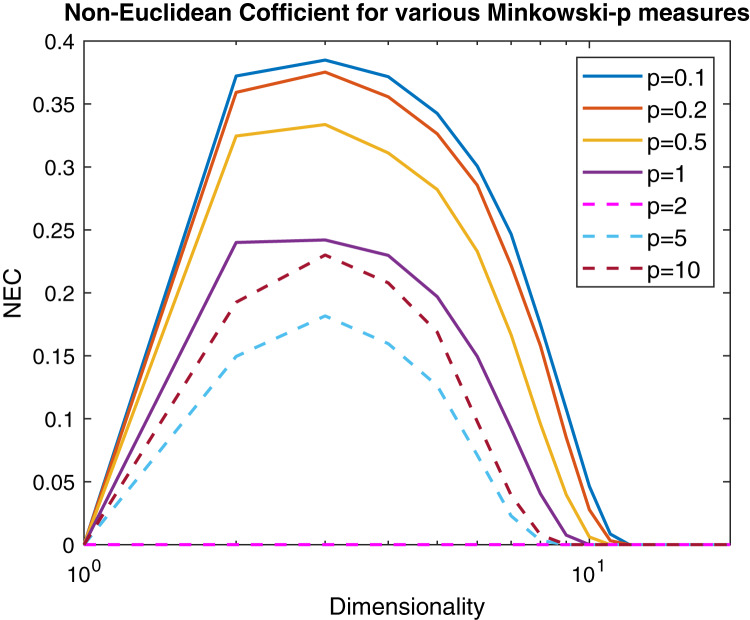
Non-Euclidean influence measured by the Negative Eigen-Fraction for several values of the *p* parameter of the Minkowski distance. Measures are computed starting from a 100-point multi-variate Gaussian distribution by varying the dimensionality.

In order to measure the non-Euclidean behavior of the space induced by the Minkowski distance, we introduce the Negative Eigen-Fraction (NEF):

(12) $$NEF = \displaystyle{{\sum\nolimits_{j = p + 1}^{p + q} \left| {{\lambda _i}} \right|} \over {\sum\nolimits_{i = 1}^{p + q} \left| {{\lambda _j}} \right|}},$$

where (*p*, *q*) is the signature of the PE space, and λ_*i*_ are the eigenvalues of the Gram matrix decomposition. The NEF measures the degree of the non-Euclidean influence evaluating the ratio between the sum of the negative eigenvalues and the overall set of eigenvalues. Another index that helps to commensurate the non-Euclidean influence is the Negative Eigen-Ratio (NER):

(13) $$NER = {r_1} = \displaystyle{{\left| {{\lambda _{min}}} \right|} \over {{\lambda _{max}}}},$$

where λ_*min*_ and λ_*max*_ are the minimum and maximum eigenvalue of the Gram matrix. In Fig. 6 are reported, following the same experimental scheme proposed in Pękalska et al. (2006), several curves representing the NEF for a 100 points Gaussian sample varying the *p* parameter of the Minkowski distance as a function of the dimensionality. Now it is clear that the Minkowski distance is non-Euclidean for any *p* ≠ 2, but for very high dimensionality values the Euclidean behavior is restored independently from *p*.

**Figure 7:**
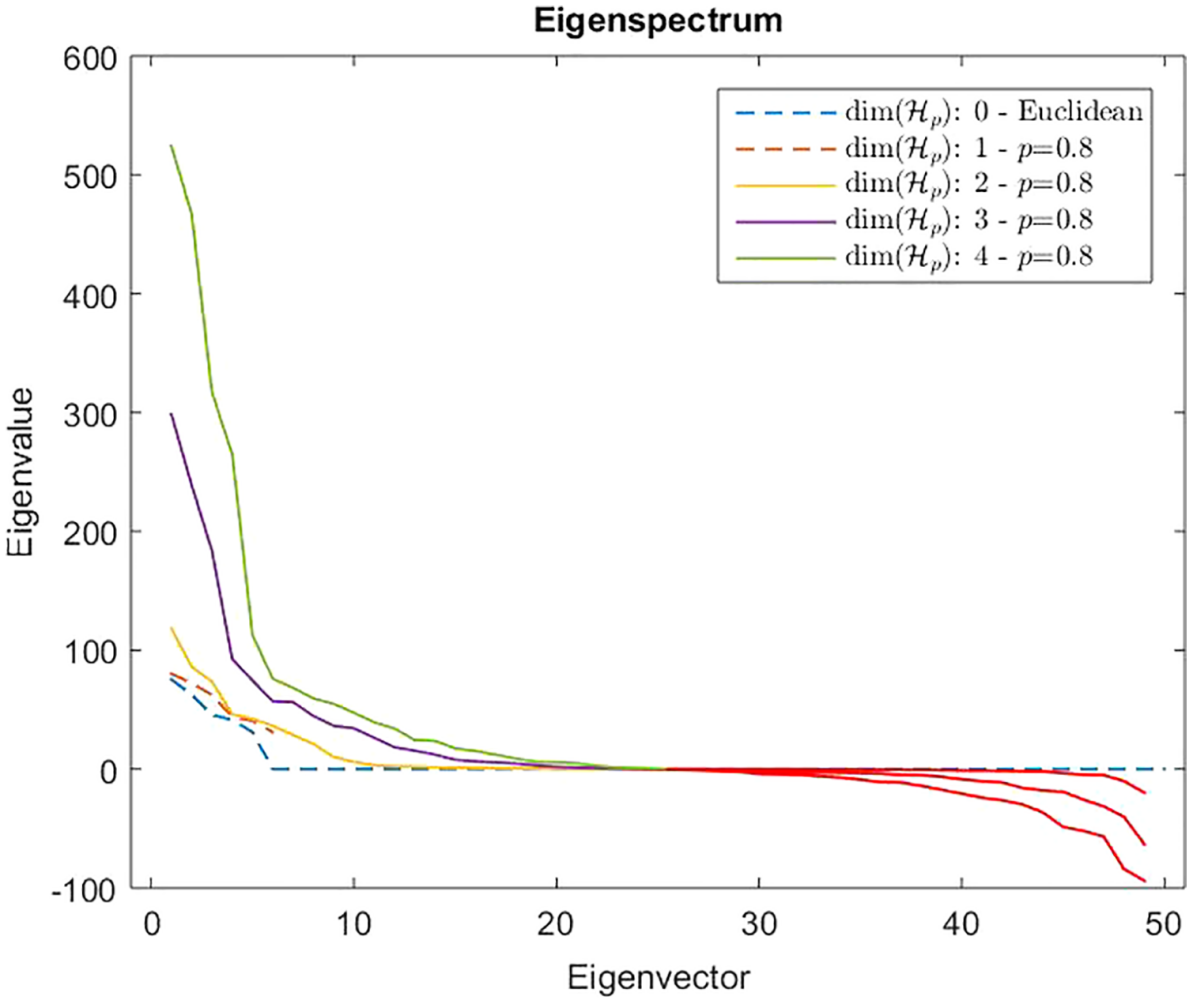
Eigenspectrum of the Gram matrix $\hat {\rm {G}}$ obtained from the dissimilarity matrix $\hat {\rm \mathcal{D}}$ computed by means of Eq. (11) in which the parameter of the Minkowski distance in *f_p_* is set as *p* = 0.8. Dashed lines show a positive eigenspectrum, while continuous lines show a mixed eigenspectrum.

**Figure 8:**
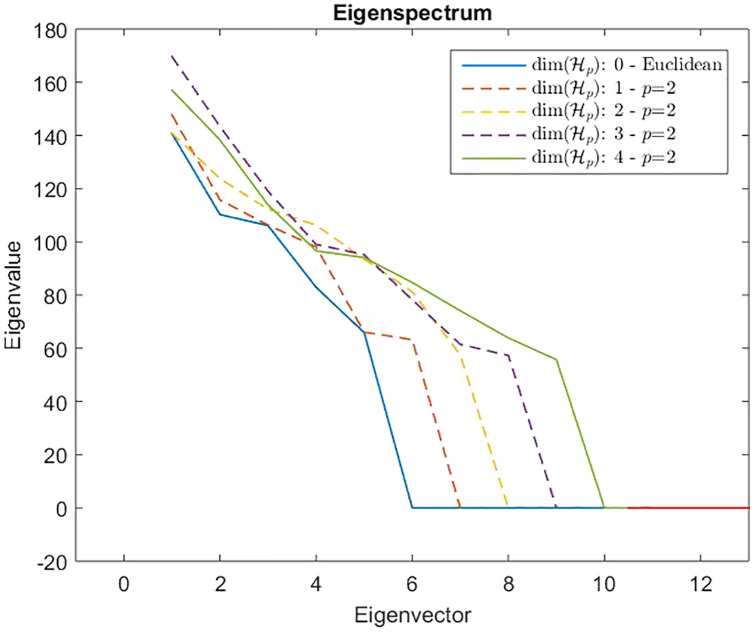
Eigenspectrum of the Gram matrix $\hat {\rm {G}}$ obtained from the dissimilarity matrix $\hat {\rm \mathcal{D}}$ computed by means of Eq. (11) in which the parameter of the Minkowski distance is set as *p* = 2, hence the standard Euclidean distance. All spectra are positive, hence the custom-based dissimilarity in Eq. (11) is Euclidean.

However, from Def. 3, we know that a *n* × *n* (*n* ≥ *m*) dissimilarity (distance) matrix ${\rm {\mathcal {D}}}$ is Euclidean if it can be embedded in a Euclidean space $\left( {{{\rm {\mathbb R}}^m},{d_2}} \right)$, where *d*_2_ is the standard Euclidean distance. It means that the Gram matrix ${\bf G}$ obtained as described in “On Metric Spaces and Dissimilarity Matrices” does not contain negative eigenvalues, hence it is a positive semi-definite matrix. The remainder of the discussion is then based on the *eigenvalues spectrum* of the Gram matrix computed from the dissimilarity matrix ${\hat {\mathcal {D}}} = {\hat d_{ij}}$. In Fig. 7 are reported the eigenvalues spectra for the Gram matrix ${\hat {\bf G}}$ obtained from the dissimilarity matrix ${\hat {\mathcal {D}}}$ computed for a fixed *k* = 5 and varying the value for *l* = 0, 1,… 4. The dashed lines are the case: *l* = 0 and *l* = 1. The first one represents the spectrum deducted from the first *k* = 5 components of ${{\rm {\mathcal {H}}}_v}$ and, as we expected, it contains only positive eigenvalues, thereby the dissimilarity matrix ${\hat {\mathcal {D}}}$ is isometrically embeddable. The same holds for *l* = 1 because trivially we have that ${f_p}(x,y) = {\left( {{{\left| {x - y} \right|}^p}} \right)^{\textstyle{1 \over p}}} = \left| {x - y} \right|,\forall x,y \in {\rm {\mathbb R}}$, thus the dissimilarity measure *f*_*p*_ remains metric. For *l* >1 the several spectra contain both positive and negative eigenvalues making the Gram matrix ${\hat {\bf G}}$ indefinite. As counterexample in Fig. 8 are depicted the spectra of the dissimilarity in Eq. (11), where the parameter *p* of the Minkowski distance is set as *p* = 2. As we expect, in this case, the dissimilarity behaves in an Euclidean fashion.

## On the presence of weights in a component-wise dissimilarity and the eigenspectrum of the gram matrix

In the discussion related to Claim 1 it is introduced a suitable component-wise custom dissimilarity that in general has the form: $d = {\left\| {{{{\bar {\bf d}}}_c}} \right\|_2} = \sqrt {{\bar {\bf d}}_c^T{{{\bar {\bf d}}}_c}}$ that is the ${\ell}$_2_ norm of the vector^14^ 14The vector **d**_*c*_ consists in the (sub)-dissimilarities that are computed on objects *x* that belongs to a suitable structured, even non-metric, dataset.

${{\bar {\bf d}}_c} = \left[ {{d_{{{\rm {\mathcal {F}}}_1}}},{d_{{{\rm {\mathcal {F}}}_2}}},...,{d_{{{\rm {\mathcal {F}}}_k}}}} \right]$ that is computed through suitable component-wise (sub)-dissimilarities, each one induced for a specific feature type ${{\rm {\mathcal {F}}}_j}$. Specifically, we had two groups: the first (sub)-dissimilarities act on a vectorial subspace and they were computed as the component-wise ${\ell}$_1_ norm: $\left| {{x_i} - {y_i}} \right|$, while in the second group a unique (sub)-dissimilarity is computed as the Minkowski distance (*p* = 0.8, hence neither metric, nor Euclidean). Now we will discuss the case in which the same family of custom-based dissimilarities are weighted, hence they have the form described in “The Weighted Euclidean Distance” for the WED. In other words, given a pair of objects *x*_*i*_ and *y*_*j*_, the dissimilarity measure under analysis has the following form:

(14) $${d_{\bf w}}\left( {{x_i},{y_j}} \right) = {\left\| {{{{\bar {\bf d}}}_c}} \right\|_{\bf w}} = \sqrt {{{{\bar {\bf d}}}_c}{{\left( {{x_i},{y_j}} \right)}^T}{{\bf W}^T}{\bf W}{{{\bar {\bf d}}}_c}\left( {{x_i},{y_j}} \right)} .$$

Given *n* objects *x*_*i*_, *i* = 1,2,…,*n*, the weighted dissimilarity matrix whose entries are given by ${d_{\bf w}}\left( {{x_i},{y_j}} \right)$–see Eq. (14)–is hereinafter referred to as ${{\rm {\mathcal {D}}}_{\bf w}}$, for convenience. The latter can be decomposed according to Eq. (1) as ${{\bf G}_{\bf w}} = - \textstyle{1 \over 2}{\rm {\mathcal {J}}{\mathcal {D}}}_{\bf w}^{*2}{\rm {\mathcal {J}}}$, where ${{\bf G}_{\bf w}}$ is the Gram matrix parametrized by the weight matrix ${\bf W}$. As discussed in “Metric Learning”, the weights act as a linear mapping ${\rm {\mathcal {M}}}:{\bf x} \to {\bf Wx}$. Starting from the above settings, two questions arise. The first is if, in principle, it is possible to find a suitable weighting matrix ${\bf W}$ that makes the dissimilarity matrix ${{\rm {\mathcal {D}}}_{\bf w}}$ “more Euclidean”. The second question is about the behavior of the Gram matrix ${{\bf G}_{\bf w}}$ in terms of eigendecomposition. In other words, one may ask what is the relationship between the eigenvalues (and eigenvectors) of the non-weighted Gram matrix ${\bf G}$ and the weighted one ${{\bf G}_{\bf w}}$.

The two questions are strongly interrelated. By the way, the first is simpler than the second. To answer the first question one may conceive a simple problem in which one wants to minimize the NEF defined in Eq. (12), hence, we can consider a diagonal matrix ${{\bf W}_{diag}} = {\rm diag}(\left[ {{w_1},{w_2},...,{w_d}} \right])$ and the task is to solve the following minimization problem:

(15) $$\matrix{ {\rm arg\;mi{n}\;{\rm NEF}({{\bf G}_{\bf w}}),} \hfill \cr {}{\bf G}_{\bf w} \cr {s.t.\;\;0 \le {w_i} \le 1\;i = 1,2,...,d.} }$$

The NEF – see Eq. (12) – depends on the eigenvalues *λ*_*i*_ of the Gram matrix which, in turn, depend on the dissimilarity matrix ${{\rm {\mathcal {D}}}_{\bf w}}$ through a non-linear operation, which in turn depends on the weighted dissimilarity measure ${d_{\bf w}}\left( {{x_i},{y_j}} \right)$, which, finally, depends on the weights matrix ${{\bf W}_{diag}}$ (if diagonal). The optimization problem can be performed *via* the same setting used to discuss Claim 1. Specifically, it is a simple exercise in adopting a meta-heuristic, such as a GA, in minimizing the optimization problem in Eq. (15). The two subspaces, ${{\rm {\mathcal {H}}}_{cw}}$ and ${{\rm {\mathcal {H}}}_v}$ have a dimensionality equal to 3 and the Minkowski parameter of the distance acting on ${{\rm {\mathcal {H}}}_{cw}}$ is set to 0.8 (hence neither metric, nor Euclidean).

Starting from a random population of 30 individuals (chromosomes) for the weights ${\bf w}$, the GA converges to the (sub)-optimal solution ${{\bf w}^*} = \left[ {1,1,0.999,0.0001} \right]$ with a fitness value (the NEF) equals to 2.0380e-06, hence negligible. As we expected, the GA finds a solution with higher weights for the “Euclidean” components and practically null value for the “Minkowski” component.

Although the answer to the first question is trivial, the second question about the relationship of the two spectra of ${\bf G}$ and ${{\bf G}_{\bf w}}$ is only apparently simple. Here we try to give a sketch of the problem. Suppose that ${\rm {\mathcal {F}}}$ is a vectorial space endowed with the standard norm $\left\langle { \cdot , \cdot } \right\rangle$, and ${\bf X} \in {{\rm {\mathbb R}}^{m \times n}}$ is a data matrix with the *n* data points organized as columns. The discussion can be restricted to an Euclidean space equipped by the standard Euclidean distance: $d\left( {{{\bf x}_i},{{\bf x}_j}} \right) = {\left\| {{{\bf x}_i} - {{\bf x}_j}} \right\|_2}$ for ${{\bf x}_i},{{\bf x}_j} \in {\bf X}$. The scalar product matrix or the Gram matrix, with the data matrix organized with data vectors in columns and the variables as rows, is: ${\bf G} = {{\bf X}^T}{\bf X}$. The linear mapping ${\rm {\mathcal {M}}}:{\bf x} \to {\bf Wx}$ transforms the data matrix ${\bf X}$ in ${\rm {\mathcal {M}}}\left( {\bf X} \right) = {\bf WX} = {\bf Y}$. Thereby, the Gram matrix becomes: ${{\bf G}_{\bf w}} = {\left( {{\bf WX}} \right)^T}\left( {{\bf WX}} \right) =  {{\bf X}^T}  {{\bf W}^T}{\bf WX} = {{\bf Y}^T}{\bf Y}$. We note that if ${\bf W}$ is invertible we have the inverse map ${{\rm {\mathcal {M}}}^{ - 1}}\left( {\bf Y} \right) =  {{\bf W}^{ - 1}}{\bf Y}$. In trying to find a relation between the eigenvalues of ${{\bf Y}^T}{\bf Y}$ and those of ${{\bf X}^T}{\bf X}$, we can make use of the relation between the Singular Value Decomposition (SVD) of a *m* × *n* matrix ${\bf A}$ and the eigendecomposition of the *n* × *n* matrix ${{\bf A}^T}{\bf A}$. In fact, any *m* × *n* matrix can be factored as ${\bf A} = {\bf U}\Sigma {{\bf V}^T}$ (Strang, 1976), where the columns of matrix ${\bf U}$ (*m* × *m*) are the eigenvectors of ${\bf A}{{\bf A}^T}$ and the columns of ${\bf V}$ (*n* × *n*) are eigenvectors of ${{\bf A}^T}{\bf A}$; finally, the $r = \rm rank\ \left( {\bf A} \right)$ singular values in the diagonal of Σ (*m* × *n*) are the square roots of the non-zero eigenvalues of both ${{\bf A}^T}{\bf A}$ and ${\bf A}{{\bf A}^T}$^15^ 15It is easy to show the relation between the eigenvalues and the singular values: **A**^*T*^ **A** = (**U**Σ**V**^*T*^)*^T^* (**U**Σ**V**^*T*^) = **V**Σ**U***^T^***U**Σ**V**^*T*^ = **V**Σ^*T*^**V**Σ^*T*^, being **U***^T^* **U** = **I**. In the same way **A**^*T*^**A** = Σ**U***^T^*Σ**U***^T^*, being **V**^*T*^**V** = **I**. **V** and **U** are orthogonal matrices for a real **A** (for complex **A** they are unitary matrices). Σ*^T^*Σ = ΣΣ*^T^* is a *n* × *n* diagonal matrix with diagonal entries the square roots of singular values of **A** that are the eigenvalues of **A**^*T*^**A** or **AA**^*T*^..

Let ${\bf Y} = {{\bf U}_{\bf w}}{\Sigma _{\bf w}}{\bf V}_{\bf w}^T$ be the SVD decomposition of ${\bf Y}$ and ${\bf X} = {\bf U}\Sigma {{\bf V}^T}$ be the decomposition of ${\bf X}$. If we multiply on the left side for ${{\bf W}^{ - 1}}$ both sides of the first relation we obtain ${{\bf W}^{ - 1}}{\bf Y} = {{\bf W}^{ - 1}}{{\bf U}_{\bf w}}{\Sigma _{\bf w}}{\bf V}_{\bf w}^T$, hence ${\bf X} = {{\bf W}^{ - 1}}{\bf Y} = {{\bf W}^{ - 1}}{{\bf U}_{\bf w}}{\Sigma _{\bf w}}{\bf V}_{\bf w}^T$. If we compare these two relations and multiply both sides for ${{\bf U}^T}$ on the left side and ${\bf V}$ on the right side and by further considering that ${{\bf V}^T}{\bf V} = {\bf I} = {{\bf U}^T}{\bf U}$, we come to the relation: ${{\bf U}^T}{\bf U}\Sigma {{\bf V}^T}{\bf V} = {{\bf U}^T}{{\bf W}^{ - 1}}{{\bf U}_{\bf w}}{\Sigma _{\bf w}}{\bf V}_{\bf w}^T{\bf V}$ that simplifies as:

(16) $$\Sigma = {{\bf U}^T}{{\bf W}^{ - 1}}{{\bf U}_{\bf w}}{\Sigma _{\bf w}}{\bf V}_{\bf w}^T{\bf V}.$$

Equation (16) is a (complex) relation between the (diagonal) singular values matrix Σ that contains as entries the singular values of ${\bf X}$ and the singular values of ${\bf Y} = {\bf WX}$, placed in the diagonal of ${\Sigma _{\bf w}}$. Unfortunately, calculations cannot be further performed in closed form unless we make additional assumption on ${\bf W}$. The reason becomes clear if we think at ${\bf WX}$ as the product of two matrices: in fact, the original question about the relationship between the eigenvalues of the Gram matrices ${\bf G}$ and ${{\bf G}_{\bf w}}$ can be translated into the relation of the eigenvalues of the following matrices ${\bf A},{\bf B},{\bf AB}$. However, this so-wanted relationship between the eigenvalues of the product of general matrices and its multiplicands is still an open problem of mathematics, even if in the literature there are a number of works that provide several inequalities for the matrix product and sum problem (Zhang & Zhang, 2006; Fulton, 2000; Watkins, 1970; Thompson & Therianos, 1971). If ${\bf W}$ is a scalar matrix of the form ${\bf W} = k{\bf I}$ the relation shown in Eq. (16) becomes simple. In fact, we can write ${\bf WX} = k{\bf IX} = {{\bf U}_{\bf w}}{\Sigma _{\bf w}}{\bf V}_{\bf w}^T$, but ${\bf X} = {\bf U}\Sigma {{\bf V}^T}$, hence ${\bf WX} = k{\bf IU}\Sigma {{\bf V}^T} =  {{\bf U}_{\bf w}}{\Sigma _{\bf w}}{\bf V}_{\bf w}^T$. It means that the singular vectors are the same: ${\bf U} = {{\bf U}_{\bf w}}$ and ${\bf V} = {{\bf V}_{\bf w}}$ and therefore Eq. (16) becomes:

(17) $$\Sigma = {k^{ - 1}}{\Sigma _{\bf w}} \Rightarrow {\Sigma _{\bf w}} = k\Sigma ,$$

Hence, for the spectrum of ${{\bf G}_{\bf w}} = {{\bf X}^T}{{\bf W}^T}{\bf WX}$, we have ${\Sigma ^T}\Sigma = {k^{ - 1}}\Sigma _{\bf w}^T{\Sigma _{\bf w}}$^16^ 16A stretching or compression transformation by a scalar matrix *k***I** leaves the eigenvectors unchanged, yet it modifies the eigenvalues..

Ultimately, there are no relationships between the spectrum of the product of two generic matrices and one of the single matrices, unless in simple cases^17^ 17It is possible to demonstrate that diagonalizable matrices share the same eigenvector matrix **S** if and only if **AB** − **BA** = 0, that is, if they commute (Strang, 1976). The result holds also for normal matrices **N**, that is, matrices where **N** commutes with **N**^*H*^ (Wilkinson, Wilkinson & Wilkinson, 1965).. In general, two generic matrices do not share the same set of eigenvectors and this makes the analysis infeasible. In order to graphically show in a computational fashion the relationship between the eigenvalues of the Gram matrix obtained from a weighted dissimilarity matrix and those obtained from a non-weighted dissimilarity matrix, we have generated a random bi-dimensional matrix ${{\bf X}^{test}} \in {{\rm {\mathbb R}}^{(2 \times 20)}}$, hence containing 20 random 2-D vectors. Moreover, the dissimilarity matrix ${\rm {\mathcal {D}}}_{\bf w}^{test}$ on ${{\bf X}^{test}}$ is computed through the standard Euclidean distance and finally the Gram matrix ${\bf G}_{\bf w}^{test}$ is extracted. The dissimilarity measure is weighted with a diagonal matrix of the form: ${\bf W} = \left[ {\matrix{ \alpha &0 \cr 0 &\beta } } \right]$, where $\alpha ,\beta \in \left( {0,1} \right]$^18^ 18We note that the eigenvalues of a diagonal matrix are the diagonal entries, *i.e*., α and β, and the eigenvectors are the canonical basis in ${\rm {\mathbb R}}^m$.. Finally the eigendecomposition of ${\bf G}_{\bf w}^{test}$ is performed, yielding the first two eigenvalues ${\lambda _{{\bf w}1}}$ and ${\lambda _{{\bf w}2}}$ as function of ${\bf W}$.

**Figure 9:**
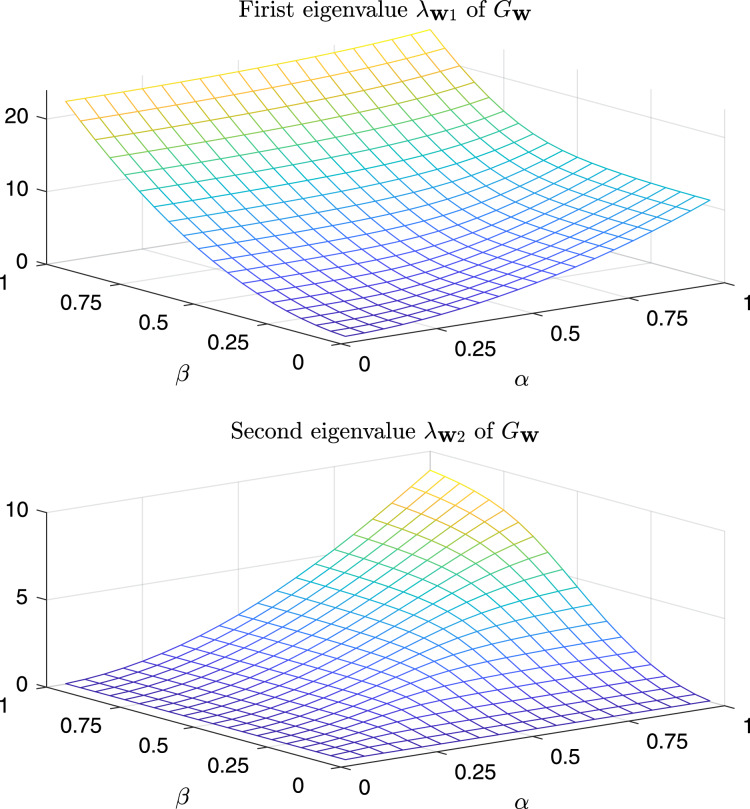
Magnitude of the first and second eigenvalue of G^*test*^_w_ as a function of *α* and *β*.

**Figure 10:**
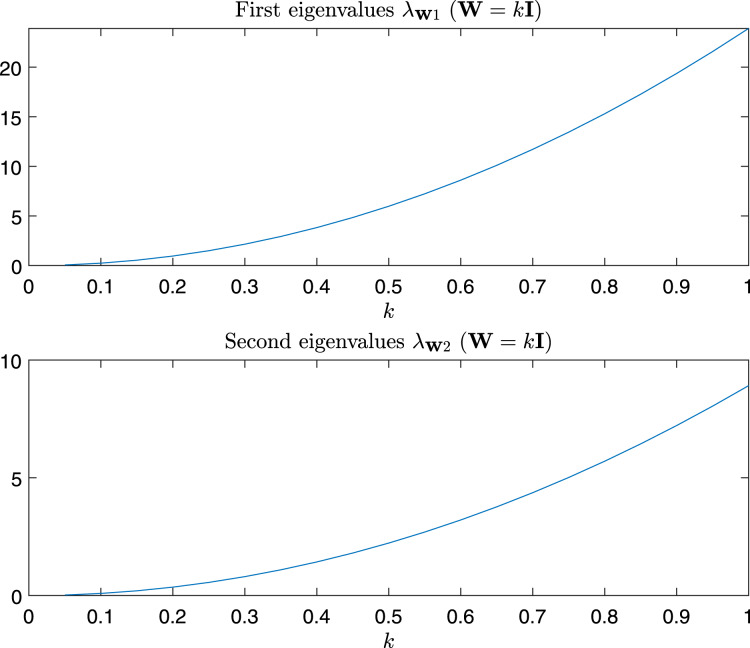
Magnitude of the first and second eigenvalue of G^*test*^_w_ in the case *α* = *β* = *k*, *i.e*., W = *k*I.

In Fig. 9 are depicted the value of the first and the second eigenvalues of ${\bf G}_{\bf w}^{test}$, respectively, as a function of *α* and *β* in the predefined interval. In Fig. 10, as instead, it is reported the value of the first and second eigenvalues in the case *α* = *β* = *k*, that is the case ${\bf W} = k{\bf I}$.

**Figure 11:**
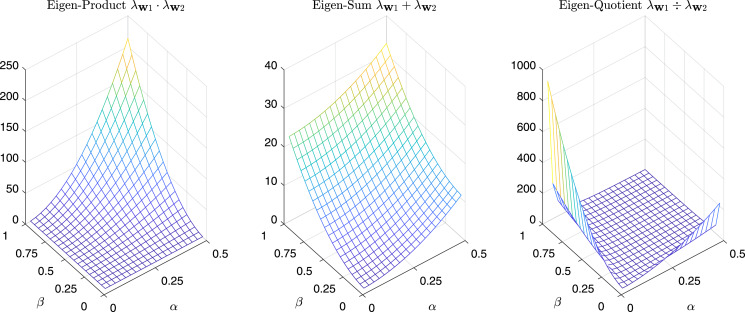
*Product, Sum* and *Quotient* of the two eigenvalues of G^*test*^_w_ as a function of *α* and *β*.

**Figure 12:**
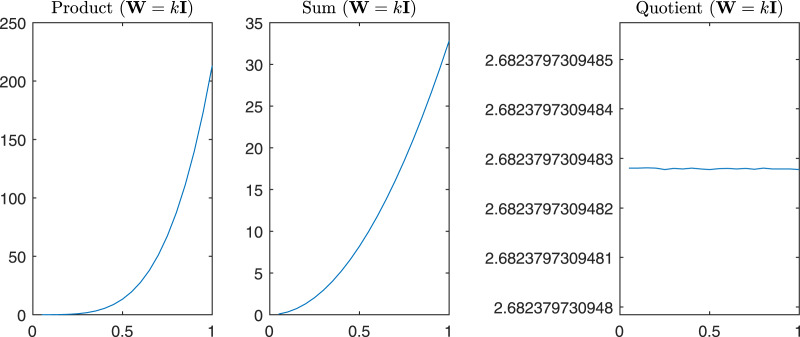
*Product, Sum* and *Quotient* of the two eigenvalues of G^*test*^_w_ in the case *α* = *β* = *k*, *i.e*., W = *k*I.

For completeness in Fig. 11 are reported the *sum*, the *product*, and the *quotient* of the first two eigenvalues of ${\bf G}_{\bf w}^{test}$, while in Fig. 12 we have the same operations in the case of *α* = *β* = *k*.

## Conclusion

In solving real-world problems in pattern recognition we may incur in a complex representation of objects with the need of a custom-based dissimilarity measure whose components are (sub)-dissimilarities tailored on the nature of the object at hand. Moreover, the starting space can be non-metric and standard machine learning algorithms cannot operate directly due to the absence of a vectorial space endowed with some well-defined norm. The dissimilarity template can be a weighted Euclidean distance where weights are learned by exploiting a metric learning paradigm. Often, in real-world applications, the adopted custom-based dissimilarity measure leads to non-Euclidean dissimilarity matrices. The non-Euclidean behavior can be suitably measured by studying the spectrum of the related Gram matrix. The adopted framework shows how the (sub)-dissimilarity measure adopted can affect the Euclidean behavior and how a weighting scheme can suitably address this phenomenon. The weighting scheme concerns the spectra of the underlying dissimilarity, but only in some simple cases the problem can be addressed theoretically. Alongside the present work of a more theoretical nature, as regards the future directions, we have planned to evaluate the impact of the non-metricity of the dissimilarity matrices in some real-world applications (*e.g*., predictive maintenance) and as a correction expressed directly in the objective function (in line with our theoretical discussion) of an optimization system impacts on the performance of a classification system in terms of generalization capabilities.

## Additional Information and Declarations

### Data Availability

The following information was supplied regarding data availability: This literature review does not contain data or code.
